# MicroRNA-129-5p Regulates Glycolysis and Cell Proliferation by Targeting the Glucose Transporter SLC2A3 in Gastric Cancer Cells

**DOI:** 10.3389/fphar.2018.00502

**Published:** 2018-05-15

**Authors:** Di Chen, Hui Wang, Jie Chen, Zhe Li, Shengli Li, Zhixiang Hu, Shenglin Huang, Yingjun Zhao, Xianghuo He

**Affiliations:** ^1^Fudan University Shanghai Cancer Center and Institutes of Biomedical Sciences, Shanghai Medical College, Fudan University, Shanghai, China; ^2^State Key Laboratory of Oncogenes and Related Genes, Shanghai Cancer Institute, Renji Hospital, Shanghai Jiao Tong University School of Medicine, Shanghai, China; ^3^Department of Oncology, Collaborative Innovation Center for Cancer Medicine, Shanghai Medical College, Fudan University, Shanghai, China

**Keywords:** miR-129-5p, gastric cancer, cancer metabolism, proliferation, SLC2A3

## Abstract

Tumor cells increase their glucose consumption through aerobic glycolysis to manufacture the necessary biomass required for proliferation, commonly known as the Warburg effect. Accumulating evidences suggest that microRNAs (miRNAs) interact with their target genes and contribute to metabolic reprogramming in cancer cells. By integrating high-throughput screening data and the existing miRNA expression datasets, we explored the roles of candidate glycometabolism-regulating miRNAs in gastric cancer (GC). Subsequent investigation of the characterized miRNAs indicated that miR-129-5p inhibits glucose metabolism in GC cells. miRNA-129-5p directly targets the 3′-UTR of SLC2A3, thereby suppressing glucose consumption, lactate production, cellular ATP levels, and glucose uptake of GC cells. In addition, the PI3K-Akt and MAPK signaling pathways are involved in the effects of the miR-129-5p/SLC2A3 axis, regulating GC glucose metabolism and growth. These results reveal a novel role of the miR-129-5p/SLC2A3 axis in reprogramming the glycometabolism process in GC cells and indicate a potential therapeutic target for the treatment of this disease.

## Introduction

Gastric cancer (GC) is the fifth most common malignancy in the world (Ferlay et al., 2015). In China, GC is the top three leading cause of cancer death among both men and women, causing approximately 679,100 new cancer cases and 498,000 deaths per year (Chen et al., 2016). Although the incidences and mortality trends for GC have declined in recent years (Siegel et al., 2014), the outcomes of this disease are still among the poorest of all solid-organ tumors, predominantly due to the frequent presence of advanced stage disease with lymphatic or distant metastasis. Current treatment strategies define curable GC as disease (stage 0–III) without distant metastasis (Shen et al., 2013), whereas stage IV disease remains incurable and carries a very poor prognosis despite the advent of molecularly targeted biological therapy and novel chemotherapy (Sonnenblick et al., 2015). As there are limited therapeutic approaches for treating advanced GC, it is urgent to elucidate new molecular mechanisms and develop more therapeutic targets for this lethal disease.

Cancer cells exhibit unrestrained growth, which are supported by metabolic adaptations that promote their survival. Aerobic glycolysis, or the Warburg effect (Warburg et al., 1927; Warburg, 1956a), a shift from oxidative phosphorylation to glycolysis and the concomitant accumulation of lactate by-products in the surrounding microenvironment, represents the best-characterized alteration of tumor cell metabolism (Gillies et al., 2008; Cairns et al., 2011b). Altered glucose metabolism is characterized by increased glucose uptake, enhanced glycolysis, and dysregulated mitochondrial oxidative phosphorylation, as well as accumulated lactate production, events that are widespread in various cancer types (Warburg, 1956a,b; Shaw, 2006; Adekola et al., 2012). Recent studies have indicated that several key rate-limiting enzymes of glycometabolic pathways are dysregulated in GC cells, contributing to increased cell proliferation and metastasis (Cui et al., 2015; Jiang et al., 2015; Shiroki et al., 2017). MicroRNAs (miRNAs) are approximately 21–25 nucleotide-long, non-coding RNA molecules that negatively regulate gene translation by binding to the 3′-untranslated region (3′-UTR) of target messenger RNAs (mRNAs), causing either enhanced mRNA degradation or inhibited protein translation (Tay et al., 2008). miRNAs have critical effects that limit the glucose metabolism of cancer cells (Fong et al., 2015; Qiu et al., 2015), and emerging evidences suggest that aberrant miRNAs participate in the altered glycometabolism and pathogenesis of GC (Liu et al., 2016; Li C.Y. et al., 2016).

In this study, we explored potential glycometabolism-regulating miRNAs in GC by integrating our previous high-throughput screening results and the existing GC miRNA expression datasets. miR-129-5p was identified as a candidate suppressor miRNA for glucose metabolism and cell proliferation, and SLC2A3 was a direct functional target gene of this miRNA in GC cells. The miR-129-5p/SLC2A3 axis may serve as a candidate therapeutic target for GC treatment.

## Materials and Methods

### Cell Culture

The HEK 293T cells were cultured in Dulbecco’s modified Eagle’s medium (DMEM; Gibco, New York City, NY, United States), and SGC-7901 and MGC-803 cells were cultured in RPMI-1640 medium (HyClone, Beijing, China). The media were supplemented with 10% fetal bovine serum (Gibco), 100 IU/mL penicillin G, and 100 μg/mL streptomycin sulfate (Sigma-Aldrich, St. Louis, MO, United States). PDGF-BB (Peprotech, Rocky Hill, CT, United States) was applied at the concentration of 50 ng/mL in RPMI-1640. Cells were cultured in a humidified 37°C incubator with 5% CO_2_.

### Transfection of Oligonucleotides

The miR-129-5p mimic (5′-CUUUUUGCGGUCUGGGCUUGC-3′) and inhibitor (5′-GCAAGCCCAGACCGCAAAAAG-3′) were designed and synthesized by Ambion (Austin, TX, United States). Three independent small interfering RNAs (siRNAs) targeting SLC2A3 were synthesized by Ribobio (Guangzhou, China), and the targeting sequences are as follows: 5′-GCTCTTTCCAATTTGGCTA-3′, 5′-CCGACAGCCCATCATCATT-3′ and 5′-GCTTCCTCATTACCTTCTT-3′. Cells were transfected with the indicated oligonucleotides or a siRNA pool (3 siRNAs were mixed in an equimolar ratio) with final concentration of 50 nM, using Lipofectamine 2000 (Life Technologies, Darmstadt, Germany). At 48 h post-transfection, cells were harvested for further detection.

### Lactate Production and Glucose Consumption

Cells were cultured in DMEM without phenol red (Gibco, New York City, NY, United States) for 15 h, and then the culture medium was collected for lactate or glucose measurement. Quantification of lactate levels was performed utilizing Lactate Assay Kit (BioVision, Mountain View, CA, United States), and glucose levels were determined by Glucose Assay Kit (Sigma-Aldrich, Cat. No. GAHK20-1KT). All values were normalized by the corresponding total protein level (BCA Protein Assay Kit; Thermo Scientific, Waltham, MA, United States).

### Cellular ATP Levels

The cellular levels of ATP were determined using CellTiter-Glo^®^ Luminescent Cell Viability Assay (Promega, Madison, WI, United States), following the manufacturer’s instructions. All values were normalized by the corresponding total protein level.

### Glucose Uptake

Cells were treated as indicated for 24 h, then seeded in a 96-well plate (13,000 cells/well) and cultured for overnight. The culture medium was removed and cells were washed with PBS. Then, cells were incubated with fresh-prepared 500 μM 2-deoxyglucose (2-DG, 50 μL/well) for 20 min at room temperature. The uptake process was stopped and neutralized, and luciferase activities were measured by Glucose Uptake-Glo Assay (Promega). Rate of glucose uptake was analyzed according to the manufacturer’s instructions.

### Colony Formation Assays, Cell Proliferation, and EdU Incorporation Assay

For colony formation assay, cells were plated in 6-well plate (1,000 cells/well) and incubated 1 week, then fixed with 4% paraformaldehyde and stained with 1% crystal violet (Sigma-Aldrich). Megascopic cell colonies were counted. Cell proliferation was measured by Cell Counting Kit-8 (CCK-8, Dojindo, Kumamoto, Japan), following the manufacturer’s instructions. For EdU incorporation assay, cells were incubated with EdU (final concentration of 10 μmol/L) for 2 h and then analyzed by Click-iT EdU Alexa Fluor^®^ Imaging Kit (Molecular Probes, Eugene, OR, United States). Images were acquired using an Olympus DP71X microscope (Olympus, Tokyo, Japan), and EdU-positive cells were counted.

### Western Blot

Proteins were separated on 7.5 or 10% sodium dodecyl sulfate-polyacrylamide gel and transferred to nitrocellulose membrane (Bio-Rad, Hercules, CA, United States). Then, the membrane was blocked with 5% non-fat milk and incubated with indicated primary antibodies. The proteins were detected using enhanced chemiluminescence reagents (Thermo Scientific). The primary antibodies used were anti-phospho-Akt (Ser473, #9271; Cell Signaling Technology, Beverly, MA, United States), anti-Akt (#9272, Cell Signaling Technology), anti-Phospho-Erk1/2 (Thr202/Tyr204, #4370, Cell Signaling Technology), anti-p44/42 MAPK (Erk1/2, #9102, Cell Signaling Technology), and anti-β-actin (60008-1-lg, Proteintech).

### RNA Extraction and Quantitative Real-Time Polymerase Chain Reaction (qPCR) Analysis

Total RNA was extracted from cells with TRIzol^®^ reagent (Invitrogen, Carlsbad, CA, United States). Reverse-transcribed complementary DNA was synthesized by PrimeScript^TM^ RT Reagent Kit (TaKaRa, Tokyo, Japan). qPCR analyses were performed with SYBR Premix Ex Taq II Kit (TaKaRa, Tokyo, Japan). The detailed primer sequences are listed in Supplementary Table S1. Mature miR129-5p were quantified with specific primers and probes using TaqMan MicroRNA Assays (Applied Biosystems, Foster City, CA, United States), with U6 small nuclear RNA as internal control.

### Vector Construction

The pri-miR-129 sequence was amplified from normal human genomic DNA and cloned into the pWPXL lentiviral vector (a generous gift from Dr. Didier Trono) to generate Lenti-miR-129-5p. pWPXL-SLC2A3 was constructed by inserting the open reading frame (ORF) of SLC2A3. The 3′-UTR sequence of SLC2A3 was amplified and inserted into the psiCHECK-2 vector (Promega, Madison, WI, United States). Detailed sequences of the primers and oligonucleotides are listed in Supplementary Table S1.

### Lentivirus Production and Transduction

Lentiviral particles were harvested 48 h after Lenti-miR-129-5p or pWPXL-SLC2A3 co-transfection with the packaging plasmid psPAX2 and the VSV-G envelope plasmid pMD2.G (psPAX2 and pMD2.G were gifts from Dr. Didier Trono) into HEK 293T cells using Lipofectamine^®^ 2000. SGC-7901 and MGC-803 cells were infected with the resultant recombinant lentivirus in the presence of 6 μg/ml polybrene (Sigma-Aldrich).

### Genome-Wide Transcriptional Profiling via the Complementary DNA (cDNA) Microarray

The SGC-7901 and MGC-803 cells were transfected with miR-129-5p mimics or control mimics. At 48 h post-transfection, cells were harvested for total RNA extraction. For microarray analysis, cells in TRIzol were shipped on dry ice to KangChen Bio-Tech (Shanghai, China) for analysis via the Agilent Whole Human Genome Oligo Microarray (one-color) platform. The RNA preparation and microarray hybridization were performed according to the manufacturer’s instructions. Differentially downregulated genes were identified through fold-change filtering (log_2_Foldchange < -1). Pathway analysis and GO analysis were used to explore the roles of these differentially expressed genes.

### Luciferase Assays

The HEK 293T cells were cultured in 96-well plates and co-transfected with 10 ng of the psiCHECK-2-SLC2A3-3′-UTR vector and either 5 pmol miR-129-5p mimics or control mimics. After 48 h of incubation, firefly and Renilla luciferase activities were measured using Dual-Luciferase Reporter Assay System (Promega).

### Statistical Analysis

Results are presented as the means ± standard error of the mean (SEM) from at least three independent experiments. Unless otherwise stated, differences between 2 groups or more than 2 groups were determined using Student’s *t*-test or one-way analysis of variance (ANOVA), respectively, followed by Dunnett’s multiple-comparisons test. *P*-values < 0.05 were considered statistically significant. Statistical analyses were performed using GraphPad Prism for Windows, version 6.00 (GraphPad Software, San Diego, CA, United States).

## Results

### miR-129-5p Is a Repressor of Glucose Metabolism in GC Cells

**FIGURE 1:**
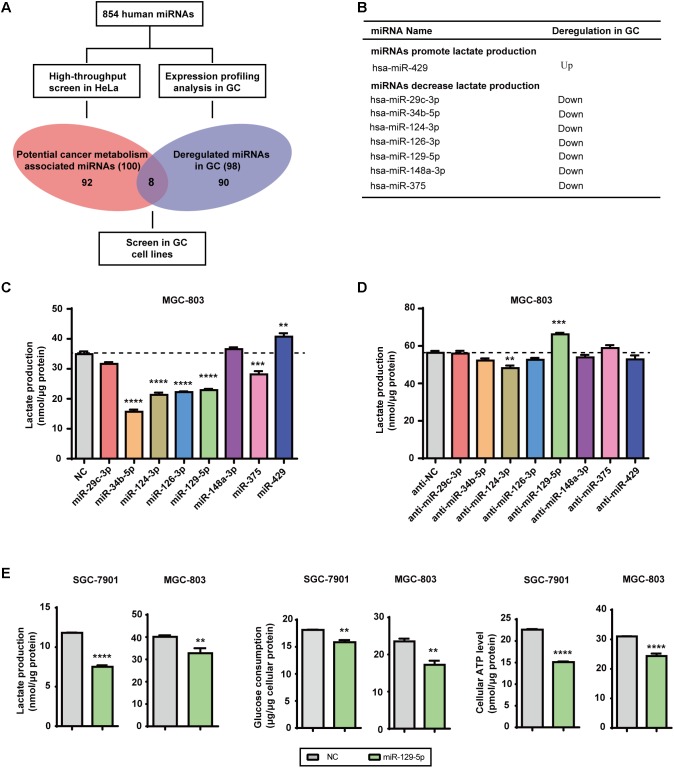
MicroRNA-129-5p is a repressor of glucose metabolism in GC cells. **(A)** A schematic diagram of the protocol used to search for candidate metabolism-associated miRNAs in GC. **(B)** Eight miRNAs were identified as candidate glycometabolism-regulating miRNAs in GC. **(C,D)** Lactate production in MGC-803 cells after transfection of the indicated miRNAs **(C)** and corresponding inhibitors **(D)**. **(E)** Lactate production, glucose consumption and cellular ATP levels in GC cells transfected with miR-129-5p mimic. Values are shown as the mean ± standard error of the mean (SEM), *n* = 3 in **(C–E)**. ^∗∗^*P* < 0.01; ^∗∗∗^*P* < 0.001; ^∗∗∗∗^*P* < 0.0001.

Lactate, the final product of aerobic glycolysis, is often overproduced in various types of tumors, which can be used to estimate the specific metabolomic characteristics and acidic microenvironment of tumor cells (Draoui and Feron, 2011). We previously identified 100 glycometabolism-regulating miRNAs using a high-throughput lactate-production screening platform in HeLa cells (Guo W. et al., 2015). By summarizing previously reported miRNA expression datasets, 98 miRNAs were selected based on their frequent dysregulation in GC tissues (Supplementary Table S2). Candidate GC-glycometabolism-related miRNAs were defined as the intersection between the 98 dysregulated miRNAs in GC and the 100 candidate glycometabolism-regulating miRNAs (**Figure 1A**). In total, seven candidate anti-metabolic miRNAs (miR-29c-3p, miR-34b-5p, miR-124-3p, miR-126-3p, miR-129-5p, miR-148a-3p, and miR-375) and one candidate pro-metabolic miRNA (miR-429) emerged (**Figure 1B**).

To further confirm the specific effects of these eight miRNAs on GC glycometabolism, we evaluated lactate production of the MGC-803 cells that were transfected with indicated miRNA mimics or miRNA inhibitors. The results showed that miR-129-5p dramatically repressed lactate production of MGC-803 cells (**Figure 1C**), whereas its inhibitor significantly elevated lactate production of MGC-803 cells (**Figure 1D**). Moreover, miR-129-5p mimics could reduce lactate production, glucose consumption and cellular ATP levels of SGC-7901 and MGC-803 cells (**Figure 1E**), indicating the potential role of miR-129-5p in GC glycometabolism. Taken together, these findings suggest that miR-129-5p inhibits glucose metabolism in GC cells.

### SLC2A3 Is the Direct Target of miR-129-5p in GC Cells

**FIGURE 2:**
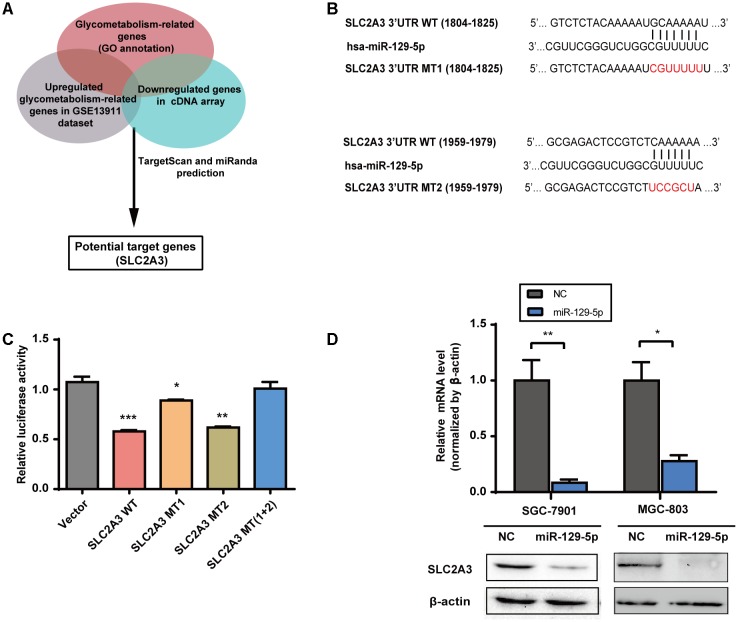
SLC2A3 is the direct target of miR-129-5p in GC cells. **(A)** Schematic representation of the strategy used to identify candidate target genes of miR-129-5p. **(B)** Diagram of putative miR-129-5p binding sites in the 3′-UTR of SLC2A3. The mutant sequences of SLC2A3 3′-UTR used in the luciferase reporter constructs are indicated in red. **(C)** Relative activities of luciferase reporters containing SLC2A3 3′-UTR variants co-transfected with miR-129-5p or negative control mimics in HEK 293T cells. **(D)** SLC2A3 mRNA and protein levels in GC cells transfected with miR-129-5p mimics. Values are shown as the mean ± SEM, *n* = 3 in **(C,D)**. ^∗^*P* < 0.05; ^∗∗^*P* < 0.01; ^∗∗∗^*P* < 0.001.

To elucidate the mechanisms underlying the inhibitory effects of miR-129-5p on the glycometabolism of GC cells, we next identified its functional target genes. The glycometabolism-related genes were clustered with the annotation of Gene Ontology Biological Function^1^. There were 29 glycometabolism-related genes that were upregulated in the GSE13911 dataset (Log_2_ FoldChange > 1, Supplementary Table S3), and 8 glycometabolism-related genes that were downregulated in miR-129-5p-treated MGC-803 cells (Log_2_ FoldChange < -1, Supplementary Table S4). Then, these genes were assessed by TargetScan^2^ prediction and miRanda^3^ prediction, and SLC2A3 was identified as a potential target of miR-129-5p responsible for GC glycometabolism (**Figure 2A**). To further validate whether SLC2A3 could be directly regulated by miR-129-5p, the wild-type (WT) or mutant (MT) 3′-UTR of SLC2A3 was introduced into luciferase reporter plasmids (**Figure 2B**). There are two predicted miR-129-5p binding sites in the 3′-UTR of SLC2A3. miR-129-5p dramatically suppressed the luciferase activity of WT SLC2A3 3′-UTR and MT2 SLC2A3 3′-UTR and had a minor effect on MT1 SLC2A3 3′-UTR, but did not affect MT (1+2) SLC2A3 3′-UTR, suggesting that miR-129-5p predominantly binds to the first predicted site (1804–1825 nt) of SLC2A3 3′-UTR (**Figure 2C**). Moreover, miR-129-5p mimic significantly reduced the mRNA and protein levels of SLC2A3 in GC cells (**Figure 2D**).

### The miR-129-5p/SLC2A3 Axis Regulates Glucose Metabolism in GC Cells

**FIGURE 3:**
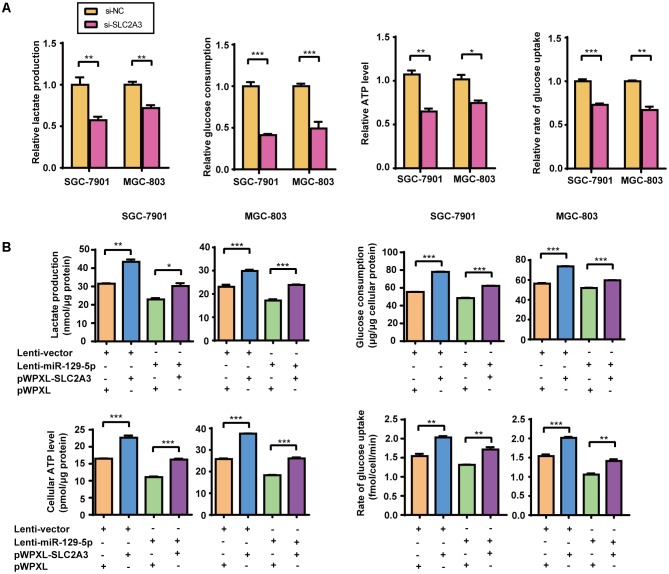
The miR-129-5p/SLC2A3 axis regulates glucose metabolism in GC cells. **(A)** SLC2A3 knockdown suppressed lactate production, glucose consumption, cellular ATP levels and glucose uptake in GC cells. **(B)** The restoration of SLC2A3 protein expression in GC cells significantly abolished the suppressive effects of miR-129-5p on lactate excretion, glucose consumption, cellular ATP levels and glucose uptake in GC cells. Values are shown as the mean ± SEM, *n* = 3. ^∗^*P* < 0.05; ^∗∗^*P* < 0.01; ^∗∗∗^*P* < 0.001.

Given that miR-129-5p represses glucose metabolism in GC cells, we next investigated the possible roles of its target gene SLC2A3 in GC glucose metabolism. Silencing of the endogenous SLC2A3 with siRNAs resulted in the dramatic suppression of the glucose consumption, lactate production, cellular ATP levels, and glucose uptake of GC cells (**Figure 3A** and Supplementary Figure S1A), which phenocopied the inhibitory effect of miR-129-5p on GC glycometabolism. Furthermore, we established SGC-7901 and MGC-803 cells with stable miR-129-5p overexpression via a lentivirus system (designated as Lenti-miR-129-5p, Supplementary Figure S1B), and constructed a lentivirus plasmid containing an SLC2A3 cDNA sequence without the 3′-UTR to reintroduce SLC2A3 into GC cells that overexpressed miR-129-5p (Supplementary Figure S1C). As expected, miR-129-5p overexpression decreased the lactate secretion, glucose consumption, cellular ATP levels, and glucose uptake of SGC-7901 and MGC-803 cells (**Figure 3B**), similar to the inhibitory effect of the specific mimics. Moreover, restoration of SLC2A3 in GC cells significantly abolished the miR-129-5p-induced suppression of lactate excretion, glucose consumption, cellular ATP levels, and glucose uptake (**Figure 3B**). These results suggest that miR-129-5p may regulate glycometabolism through SLC2A3 expression in GC cells.

### The miR-129-5p/SLC2A3 Axis Regulates the Proliferation of GC Cells

**FIGURE 4:**
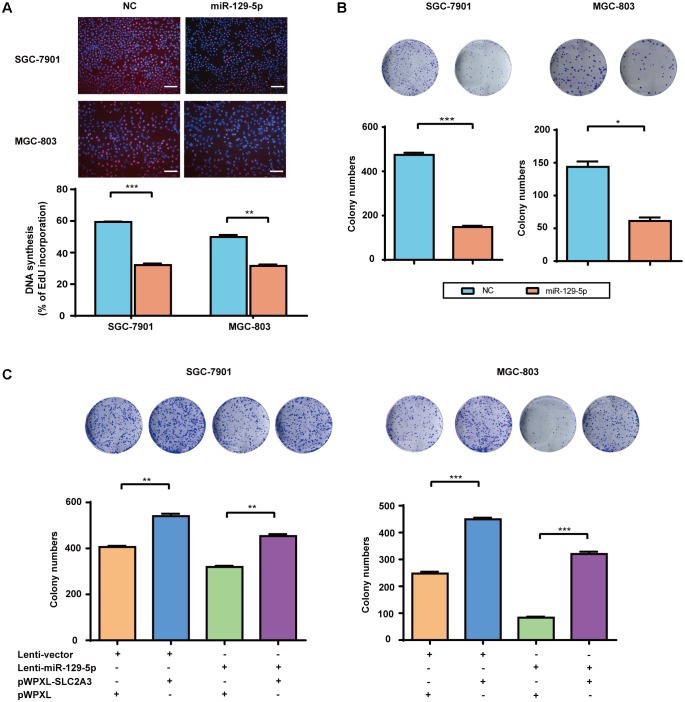
The miR-129-5p/SLC2A3 axis regulates the proliferation of GC cells. **(A)** miR-129-5p suppressed DNA synthesis in GC cells. *Upper:* Representative images of EdU assays (the scale represents 200 μm, original magnification × 200). *Lower:* Quantification of the EdU incorporation rate in GC cells. **(B)** miR-129-5p mimics inhibited GC cell colony formation. *Upper:* Representative images. *Lower:* Quantification of colony numbers. **(C)** The reintroduction of SLC2A3 significantly reversed miR-129-5p-induced inhibition of cell colony formation. *Upper:* Representative images. *Lower:* Quantification of colony numbers. Values are shown as the mean ± SEM, *n* = 3. ^∗^*P* < 0.05; ^∗∗^*P* < 0.01; ^∗∗∗^*P* < 0.001.

Over the recent decade, advanced studies of cancer metabolism have broadened our understanding of how disturbed glucose metabolism can be involved in carcinogenesis (Ward and Thompson, 2012). miR-129-5p was previously identified as a glycometabolism-related miRNA in GC, then we further characterized its effects on the proliferation of GC cells. EdU incorporation assays showed that miR-129-5p mimic treatment significantly inhibited the proliferation of SGC-7901 and MGC-803 cells (**Figure 4A**). Moreover, miR-129-5p mimic treatment suppressed the colony formation ability of GC cells (**Figure 4B**). These results demonstrated that miR-129-5p might act as a suppressor miRNA in GC carcinogenesis. Indeed, miR-129-5p was significantly downregulated in GC tissues in the TCGA cohort (Supplementary Figure S2), compared with that in non-tumor tissues.

Regarding the function of miR-129-5p/SLC2A3 axis in regulating GC glycometabolism, we then examined whether this axis might contribute to the proliferation of GC cells. As shown in **Figure 4C**, Lenti-129-5p treatment significantly inhibited the colony formation abilities of SGC-7901 and MGC-803 cells, whereas SLC2A3 overexpression dramatically enhanced the colony formation abilities of these cells. Importantly, ectopic expression of SLC2A3 overcame the anti-proliferation effects of miR-129-5p in GC cells (**Figure 4C**), indicating that targeting SLC2A3 is an important mechanism for the anti-proliferation function of miR-129-5p.

### miR-129-5p Reprograms Gene Expression Profiling in GC Cells

**FIGURE 5:**
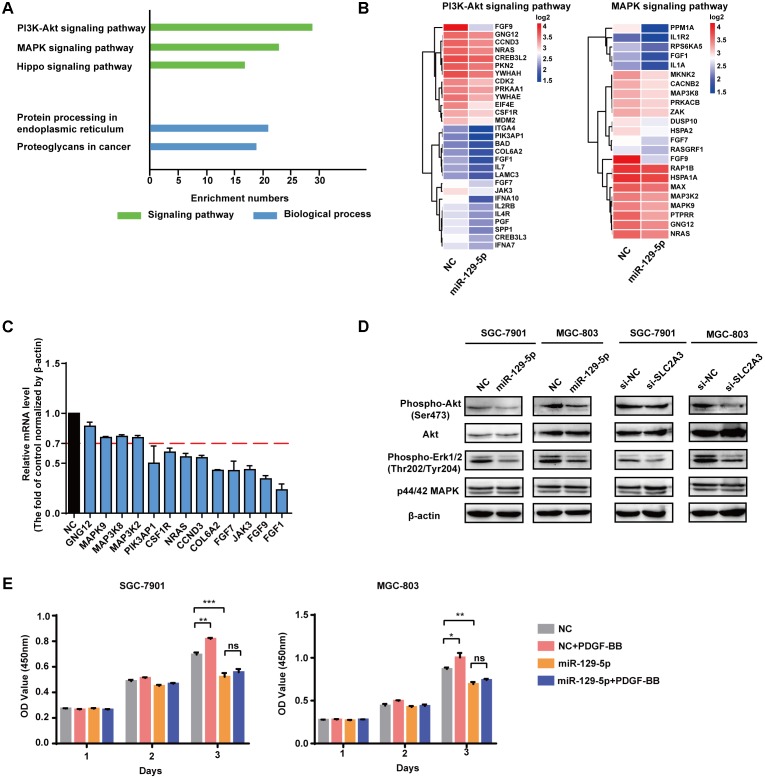
miR-129-5p reprograms gene expression profiling in GC cells. **(A)** Functional gene annotation clustering of genes regulated by miR-129-5p in MGC-803 cells. Significantly enriched groups are ranked based on the group enrichment scores, suggested by the gene ontology terms. Green, signaling pathway. Blue, biological process. **(B)** Expression levels of the subsetting genes involved in PI3K-Akt signaling pathway and MAPK signaling pathway. The genes are shaded with blue or red in the heatmap to indicate low or high expression, respectively. **(C)** qPCR analysis for the selected genes from ranked pathways of MGC-803 cells transfected with miR-129-5p mimics or negative controls. **(D)** Western blotting assays for PI3K-Akt and MAPK signaling pathways in SGC-7901 and MGC-803 cells transfected with miR-129-5p mimics or SLC2A3 siRNAs. **(E)** CCK8 assays for SGC-7901 and MGC-803 cells transfected with miR-129-5p mimics, with or without PDGF-BB treatment. Values are shown as the mean ± SEM, *n* = 3 in **(C,E)**. ^∗^*P* < 0.05; ^∗∗^*P* < 0.01; ^∗∗∗^*P* < 0.001; ns, not significant. β-actin served as internal control.

To identify the molecular processes and signaling pathways underlying the suppressor activity of miR-129-5p in gastric glycometabolism and carcinogenesis, the gene expression profiling of miR-129-5p-treated MGC-803 cells was analyzed by cDNA microarrays. Functional annotation revealed that signaling pathway gene sets that primarily affected by miR-129-5p include PI3K-Akt signaling pathway, MAPK signaling pathway and Hippo signaling pathways, resulting in the remarkable changes in protein processing in the endoplasmic reticulum and proteoglycans process in cancer (**Figure 5A**). The cDNA microarray also confirmed that genes in the top-scoring processes, such as those in PI3K-Akt signaling pathway and MAPK signaling pathway (**Figure 5B**), could be regulated by miR-129-5p in MGC-803 cells (**Figure 5C**). Furthermore, miR-129-5p could inhibit the phosphorylation levels of Akt and Erk1/2 in GC cells without obvious changes in the total levels of these proteins, and SLC2A3 knockdown by specific siRNAs suppressed Akt and Erk1/2 phosphorylation (**Figure 5D**), similar to the effects of miR-129-5p overexpression in GC cells. Moreover, when SGC-7901 and MGC-803 cells were treated with only PDGF-BB, introduction of miR-129-5p in these cells remarkably abolished the promoting effects of PDGF-BB on GC cell proliferation (**Figure 5E**). These results suggest that the miR-129-5p/SLC2A3 axis exerts its biological activities on glycometabolism and carcinogenesis primarily through modulating the PI3K-Akt and MAPK signaling pathways in GC cells.

## Discussion

Emerging evidences suggest that aberrant miRNAs participate in the pathogenesis of GC (Guo L.L. et al., 2015). Recently, several miRNAs, such as miR-let-7a, miR-148b and miR-181b, were identified as regulators of glycolysis and the citric acid cycle in GC (Tang et al., 2016; Li L.Q. et al., 2016; Ding et al., 2017). We previously identified 100 glycometabolism-regulating miRNAs from an 854-miRNA library using a high-content screening procedure (Guo W. et al., 2015). In the present study, we demonstrated that miR-129-5p could dramatically repress lactate production, glucose consumption, cellular ATP levels, and glucose uptake in GC cells. miR-129 was previously implicated in gastrointestinal cancer (Fesler et al., 2014), due to targeting important genes that are associated with tumorigenesis, disease progression, cell cycle, cell motility, and chemoresistance. The level miR-129-5p was found to be significantly decreased in GC and positively associated with overall survival of GC patient (Li C.Y. et al., 2016). In addition to targeting coding genes, miR-129-5p contributed to lncRNA-AC130710 upregulation in GC tissues (Xu et al., 2014). Hypermethylation of a miR-129-5p CpG island might play important roles in the development of GC chemoresistance (Wu et al., 2014). As expected, ectopic expression of miR-129-5p significantly inhibited the colony formation abilities and the growth of GC cells. Our findings highlight an additional mechanism for miR-129-5p-inhibited cell growth, which is mediated by the regulation of glucose metabolism in GC cells.

Tumor cells increase their glucose consumption to generate the necessary biomass for their proliferation (Cairns et al., 2011a), and they upregulate facilitative glucose transporter (GLUT) proteins to achieve sufficient glucose uptake (Barron et al., 2016). GLUT proteins, encoded by the *SLC2* genes, are members of the major facilitator superfamily of membrane transporters (Mueckler and Thorens, 2013), and responsible for the first step of cellular glucose utilization by facilitative diffusion of glucose (Masin et al., 2014). SLC2A3 (GLUT3), a transporter with a high affinity for glucose (Km approximately 1.5 mM) and the highest calculated glucose turnover rate, transports glucose across the cell membrane in an energy-independent manner (Rodriguez-Enriquez et al., 2009). Including GC, positive staining results for SLC2A3 have been detected in several malignant tumor tissues (Younes et al., 1997), suggesting that SLC2A3 may participate in facilitating glucose uptake in the tumors with intense glucose requirements. Previous studies have shown that SLC2A3 could be directly regulated by miR-106a in glioblastoma (Dai et al., 2013), as well as by miR-195-5p in bladder cancer (Fei et al., 2012). In this study, we demonstrate that SLC2A3 is a direct functional target of miR-129-5p in GC cells and the miR-129-5p/SLC2A3 axis plays important roles in the reprogramming of glycometabolism for tumor bioenergetics and biosynthesis, thus influencing GC cell growth.

Our study further shows that PI3K-Akt signaling pathway and MAPK signaling pathway are involved in mediating the effects of miR-129-5p in GC cells. Gene Set Enrichment Analysis (GSEA) revealed that these two signaling pathways were the top-scoring processes affected by miR-129-5p in GC cells. As Akt and MAPK are pivotal growth-promoting signaling modulators (Wilhelm et al., 2004; Garcia et al., 2006), it is not surprising that they contribute to the proliferation of GC cells as downstream effectors of many stimuli (Wang et al., 2012; Ye et al., 2016). However, the extensive interplay between these pathways and miRNAs, that facilitates the Warburg effect and cell growth in GC cells, is still poorly understood. Our results revealed that the glycometabolism-related miR-129-5p/SLC2A3 axis could significantly affect the phosphorylation of Akt and ERK1/2, and miR-129-5p could erase the promoting effects of PDGF-BB on GC cell proliferation, indicating that PI3K-Akt and MAPK signaling pathways may be implicated in the regulated GC glycometabolism and cell growth by the miR-129-5p/SLC2A3 axis.

## Conclusion

We demonstrate that miR-129-5p is a vital regulator of glucose metabolism and cell proliferation in GC cells, and SLC2A3 is the direct functional target of this miRNA. The newly identified miR-129-5p/SLC2A3 axis plays an essential role in GC glucose metabolism and cell growth through the involvement of the PI3K-Akt and MAPK pathways. Our observations provide new insights into the pathogenesis of GC and suggest a novel potential therapeutic target for the treatment of this disease.

## Ethics Statement

Before the experiments, all procedures were approved by the ethical committee of the Fudan University.

## Author Contributions

XH, YZ, DC, and HW conceived of the presented idea. DC and HW carried out the experiment. DC wrote the manuscript with support from XH, YZ, and HW. DC, YZ, HW, SL and SH analyzed the data. All authors discussed the results and contributed to the final manuscript.

## Conflict of Interest Statement

The authors declare that the research was conducted in the absence of any commercial or financial relationships that could be construed as a potential conflict of interest. The reviewer XT and handling Editor declared their shared affiliation.

## Footnotes

1 http://geneontology.org/

2 http://www.targetscan.org

3 http://www.microrna.org

## Abbreviations

3′-UTR: 3′-untranslated region
GC: gastric cancer
GSEA: Gene Set Enrichment Analysis
SLC2A3: solute carrier family 2 member 3
TCGA: The Cancer Genome Atlas
