# Supplementary material for: MicroRNA-129-5p Regulates Glycolysis and Cell Proliferation by Targeting the Glucose Transporter SLC2A3 in Gastric Cancer Cells
Source: Front Pharmacol. 2018 May 15;9:502. doi: 10.3389/fphar.2018.00502 (PMC5962750; doi:10.3389/fphar.2018.00502)
Supplement: Supplementary file 1 [file Data_Sheet_1.doc]

**Supplementary data**

**MicroRNA-129-5p regulates glycolysis and cell proliferation by targeting glucose transporter SLC2A3 in gastric cancer cells**

Di Chen^1^*, Hui Wang^2^*, Jie Chen^1^, Zhe Li^1^, Shengli Li^1^, Zhixiang Hu^1^, Shenglin Huang^1^, Yingjun Zhao^1, †^, Xianghuo He^1, 3, †^

^1^Fudan University Shanghai Cancer Center and Institutes of Biomedical Sciences; Department of Oncology, Shanghai Medical College, Fudan University, Shanghai 200032, China;

^2^State Key Laboratory of Oncogenes and Related Genes, Shanghai Cancer Institute, Renji Hospital, Shanghai Jiao Tong University School of Medicine, Shanghai 200032, China;

^3^Collaborative Innovation Center for Cancer Medicine, Department of Oncology, Shanghai Medical College, Fudan University, Shanghai 200032, China.

*These authors contributed equally to this work.

^†^ Corresponding Author: [xhhe@fudan.edu.cn](mailto:xhhe@fudan.edu.cn) (X. H.), [zhaoyingjun@fudan.edu.cn](mailto:zhaoyingjun@fudan.edu.cn) (Y. Z.) Fudan University Shanghai Cancer Center and Institutes of Biomedical Sciences; Shanghai Medical College, Fudan University, 1201 Rm., 2# Bldg., 270 Dong An Road, Shanghai 200032, China. Tel: 86-21-34777577; Fax: 86-21-64172585.

**Supplementary Figures**

**
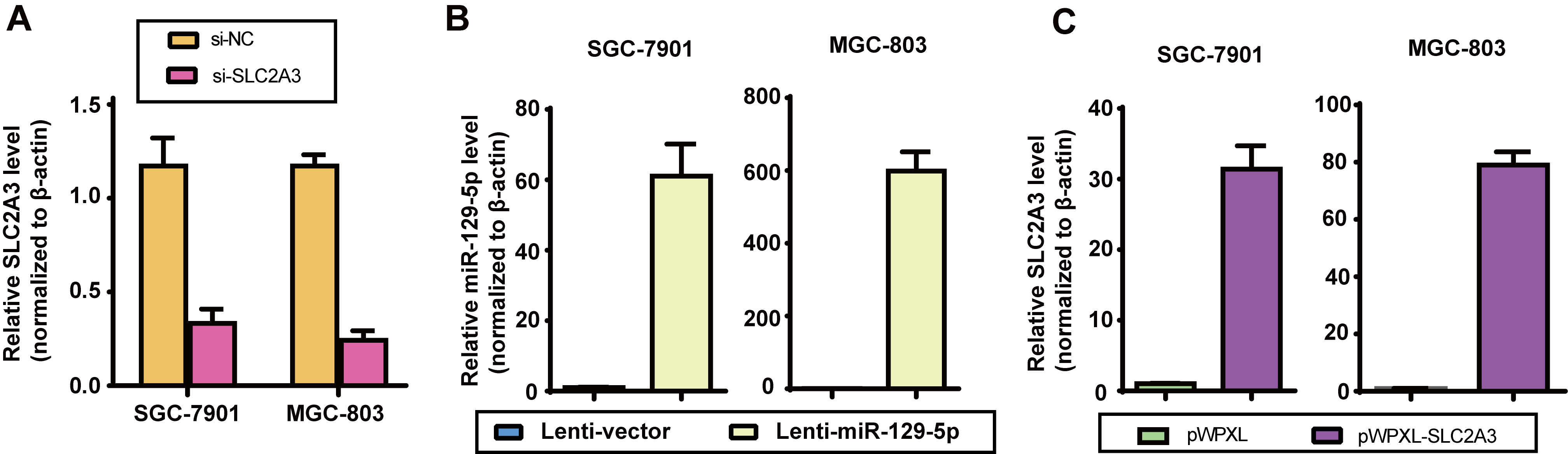
**

**Figure S1. The expression levels of SLC2A3 or miR-129-5p in the indicated cells.** (A) SLC2A3 expression levels were evaluated in SGC-7901 and MGC-803 cells after transfection with SLC2A3 small interfering RNAs for 48 h. (B) miR-129-5p expression levels were evaluated in SGC-7901 and MGC-803 cells that stably over-expressed miR-129-5p via lenti-virus. (C) SLC2A3 expression levels were evaluated in SGC-7901 and MGC-803 cells that stably over-expressed SLC2A3 via lenti-virus. Values are shown as the mean ± SEM, n=3. ***P < 0.001.


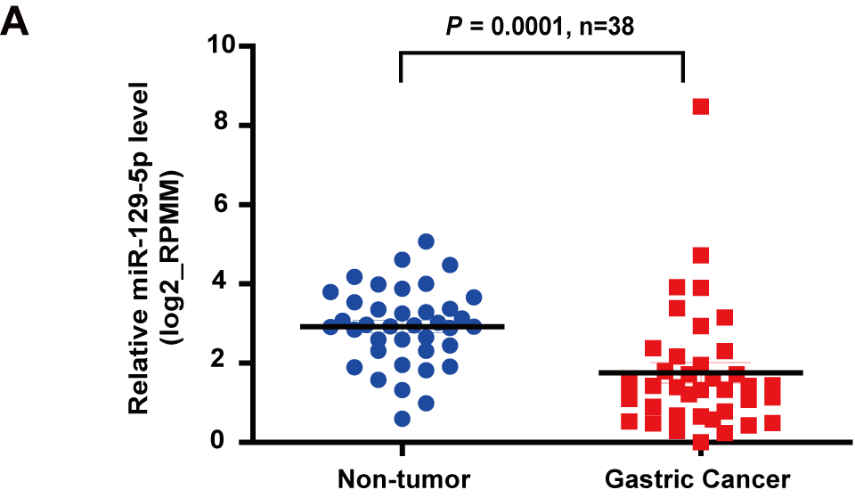


**Figure S2. The expression levels of miR-129-5p in 38 paired TCGA GC and adjacent non-tumour samples.** Values are expressed as the median with interquartile range, n=38.
