# Supplementary material for: MicroRNA-129-5p Regulates Glycolysis and Cell Proliferation by Targeting the Glucose Transporter SLC2A3 in Gastric Cancer Cells
Source: Front Pharmacol. 2018 May 15;9:502. doi: 10.3389/fphar.2018.00502 (PMC5962750; doi:10.3389/fphar.2018.00502)
Supplement: Supplementary file 2 [file Table_1.doc]

**Supplementary Table**

**MicroRNA-129-5p regulates glycolysis and cell proliferation by targeting glucose transporter SLC2A3 in gastric cancer cells**

Di Chen^1^*, Hui Wang^2^*, Jie Chen^1^, Zhe Li^1^, Shengli Li^1^, Zhixiang Hu^1^, Shenglin Huang^1^, Yingjun Zhao^1, †^, Xianghuo He^1, 3, †^

^1^Fudan University Shanghai Cancer Center and Institutes of Biomedical Sciences; Department of Oncology, Shanghai Medical College, Fudan University, Shanghai 200032, China;

^2^State Key Laboratory of Oncogenes and Related Genes, Shanghai Cancer Institute, Renji Hospital, Shanghai Jiao Tong University School of Medicine, Shanghai 200032, China;

^3^Collaborative Innovation Center for Cancer Medicine, Department of Oncology, Shanghai Medical College, Fudan University, Shanghai 200032, China.

*These authors contributed equally to this work.

^†^ Corresponding Author: [xhhe@fudan.edu.cn](mailto:xhhe@fudan.edu.cn) (X. H.), [zhaoyingjun@fudan.edu.cn](mailto:zhaoyingjun@fudan.edu.cn) (Y. Z.) Fudan University Shanghai Cancer Center and Institutes of Biomedical Sciences; Shanghai Medical College, Fudan University, 1201 Rm., 2# Bldg., 270 Dong An Road, Shanghai 200032, China. Tel: 86-21-34777577; Fax: 86-21-64172585.

**Supplementary Tables**

**Table S1. Sequences for primers**.

| **Name** | **Sequence (5' to 3')** |
| --- | --- |
| **qPCR primers** |  |
| SLC2A3-q-F | GGTCGCTTGGTTATTGGC |
| SLC2A3-q-R | ACCGCTGGAGGATCTGCT |
| SLC2A1-q-F | CTTCACTGTCGTGTCGCTGT |
| SLC2A1-q-R | GATGGCCACGATGCTCAGAT |
| SLC2A2-q-F | CCGACAGCCTATTCTAGTGGCATTG |
| SLC2A2-q-R | TGTTGATGGCACCAACTCCGATG |
| GNG12-q-F | ACTCCTGGCATGTTCCTCAC |
| GNG12-q-R | CAATATAGCCCAGGCAAGGA |
| MAPK9-q-F | GCTCTGCGTCACCCATACAT |
| MAPK9-q-R | GCTGCATCTGAAGGCTGATCT |
| MAP3K8-q-F | GCCAAGGCCGCAGATGCAAT |
| MAP3K8-q-R | TGCAGTGTCAGGGGCATGGT |
| MAP3K2-q-F | GACCAACTGGAGATTGGGCA |
| MAP3K2-q-R | TCCTTAATTGAACCCCCTGGC |
| PIK3AP1-q-F | AAGAGGAACTGATGCACGGG |
| PIK3AP1-q-R | TTCCCATGCTGTTGCCTTCT |
| CSF1R-q-F | TGCGGCCAGGCTAAAAGG |
| CSF1R-q-R | GGGACACTGGGCTCTATCAC |
| NRAS-q-F | GTGGAGCTTGAGGTTCTTGCT |
| NRAS-q-R | CCTTCGCCTGTCCTCATGTA |
| CCND3-q-F | CTGGCCATGAACTACCTGGA |
| CCND3-q-R | CCAGGAAATCATGTGCAATC |
| COL6A2-q-F | CTCGGGACCAGGACTTCAG |
| COL6A2-q-R | GTAGTGTCCGGCGAGATGAC |
| FGF7-q-F | TCTTATATATCCAGCTGTTAGC |
| FGF7-q-R | AACAGTTCGTAGTAAGTTCAG |
| JAK3-q-F | CCTGGATCCTGCTAAGAAACTCC |
| JAK3-q-F | CATTCCACAGCCCATCACGA |
| FGF9-q-F | TGGACAGCCCGGTTTTGTTA |
| FGF9-q-R | TTCCAGAATGCCAAATCGGCT |
| FGF1-q-F | AAGCCCGTCGGTGTCCATGG |
| FGF1-q-R | GATGGCACAGTGGATGGGAC |
| β-actin-q-F | TTGTTACAGGAAGTCCCTTGCC |
| β-actin-q-R | ATGCTATCACCTCCCCTGTGTG |
| **Primers for cloning** |  |
| SLC2A3-3'UTR-F | CACTATTTAGGAGCCTACGT |
| SLC2A3-3'UTR-R | TGTCGTGTACATTCAGAATT |
| SLC2A3-ORF-F | CGGGATCCCG ATGGGGACACAGAAGGTC |
| SLC2A3-ORF-R | GGACTAGTCC TTAGACATTGGTGGTGGT |

**Table S2.** Deregulated miRNAs in GC.

| **miRNA Name** | **Deregulation** | **References** |
| --- | --- | --- |
|
| hsa-miR-15a-5p | up | 1 |
| hsa-miR-16-5p | up | 1 |
| hsa-miR-21-5p | up | 3 |
| hsa-miR-23a | up | 1 |
| hsa-miR-25-3p | up | 1 |
| hsa-miR-27a | up | 1 |
| hsa-miR-103 | up | 1 |
| hsa-miR-103a-3p | up | 2 |
| hsa-miR-106a-5p | up | 2 |
| hsa-miR-106b-5p | up | 1 |
| hsa-miR-107 | up | 1 |
| hsa-miR-130b-3p | up | 2 |
| hsa-miR-135b-5p | up | 2 |
| hsa-miR-144-3p | up | 2 |
| hsa-miR-146a-5p | up | 1 |
| hsa-miR-150-5p | up | 2 |
| hsa-miR-151a-3p | up | 2 |
| hsa-miR-181a-5p | up | 2 |
| hsa-miR-181a-5p | up | 2 |
| hsa-miR-181d | up | 2 |
| hsa-miR-183-5p | up | 2 |
| hsa-miR-196a-5p | up | 1 |
| hsa-miR-196b-5p | up | 1 |
| hsa-miR-199a-5p | up | 1 |
| hsa-miR-200a-3p | up | 1 |
| hsa-miR-214-3p | up | 1 |
| hsa-miR-215 | up | 1 |
| hsa-miR-221-3p | up | 3 |
| hsa-miR-222-3p | up | 3 |
| hsa-miR-223-3p | up | 3 |
| hsa-miR-224-5p | up | 3 |
| hsa-miR-320a | up | 1 |
| hsa-miR-372 | up | 1 |
| hsa-miR-379-5p | up | 1 |
| hsa-miR-421 | up | 1 |
| hsa-miR-429 | up | 1 |
| hsa-miR-545-3p | up | 3 |
| hsa-miR-550a-3p | up | 3 |
| hsa-miR-650 | up | 1 |
| hsa-let-7a-5p | down | 1 |
| hsa-let-7b-5p | down | 2 |
| hsa-let-7c | down | 1 |
| hsa-let-7d-5p | down | 2 |
| hsa-let-7e-5p | down | 2 |
| hsa-let-7f-5p | down | 1 |
| hsa-let-7g-5p | down | 1 |
| hsa-let-7i-5p | down | 2 |
| hsa-miR-1 | down | 2 |
| hsa-miR-9-5p | down | 1 |
| hsa-miR-10b | down | 1 |
| hsa-miR-29c-3p | down | 1 |
| hsa-miR-30e-3p | down | 3 |
| hsa-miR-31-5p | down | 1 |
| hsa-miR-34b-5p | down | 1 |
| hsa-miR-43c | down | 1 |
| hsa-miR-95 | down | 2 |
| hsa-miR-101-3p | down | 1 |
| hsa-miR-124-3p | down | 1 |
| hsa-miR-125a-5p | down | 1 |
| hsa-miR-126-3p | down | 1 |
| hsa-miR-129-5p | down | 1 |
| hsa-miR-130a-3p | down | 3 |
| hsa-miR-137 | down | 1 |
| hsa-miR-139-5p | down | 1 |
| hsa-miR-141-3p | down | 1 |
| hsa-miR-148a-3p | down | 3 |
| hsa-miR-148b-3p | down | 1 |
| hsa-miR-152 | down | 1 |
| hsa-miR-154-5p | down | 3 |
| hsa-miR-155-5p | down | 1 |
| hsa-miR-181b-5p | down | 1 |
| hsa-miR-181c-5p | down | 1 |
| hsa-miR-193b-3p | down | 3 |
| hsa-miR-203a | down | 1 |
| hsa-miR-204-5p | down | 1 |
| hsa-miR-212-3p | down | 1 |
| hsa-miR-218-5p | down | 1 |
| hsa-miR-299-3p | down | 3 |
| hsa-miR-323b-5p | down | 3 |
| hsa-miR-331-3p | down | 1 |
| hsa-miR-335-5p | down | 1 |
| hsa-miR-363-3p | down | 2 |
| hsa-miR-375 | down | 1 |
| hsa-miR-377-3p | down | 3 |
| hsa-miR-433 | down | 1 |
| hsa-miR-449a | down | 1 |
| hsa-miR-451a | down | 1 |
| hsa-miR-486-5p | down | 1 |
| hsa-miR-490-3p | down | 3 |
| hsa-miR-497-5p | down | 1 |
| hsa-miR-512-5p | down | 1 |
| hsa-miR-622 | down | 1 |
| hsa-miR-652-3p | down | 1 |
| hsa-miR-661 | down | 3 |
| hsa-miR-668 | down | 2 |
| hsa-miR-768-3p | down | 3 |
| hsa-miR-768-5p | down | 3 |
| hsa-miR-770-5p | down | 2 |

**Table S3.** Glycometabolism-related genes up-regulated in GC.

| **GeneSymbol** | **Deregulation** |
| --- | --- |
|
| *GALM* | up |
| *G6PC3* | up |
| *ACLY* | up |
| *BPGM* | up |
| *C12orf5* | up |
| *DLAT* | up |
| *ENO1* | up |
| *ENO2* | up |
| *GAPDH* | up |
| *GPI* | up |
| *HK3* | up |
| *IDH3A* | up |
| *IDH3B* | up |
| *LDHA* | up |
| *MDH2* | up |
| *PDK3* | up |
| *PFKP* | up |
| *PGAM1* | up |
| *PGK1* | up |
| *PGM2* | up |
| *PRPS1* | up |
| *RPE* | up |
| *RPIA* | up |
| *SDHC* | up |
| *SLC2A1* | up |
| *SLC2A3* | up |
| *TALDO1* | up |
| *TKT* | up |
| *TPI1* | up |

**Table S4. Glycometabolism-related genes downregulated by miR-129-5p mimic in MGC-803 cells.**

| **GeneSymbol** | **Deregulation** | **Signal Intensities (FPKM)** | | **Log_2_ FoldChange**  **(miR-129-5p/NC)** |
| --- | --- | --- | --- | --- |
|  |  | **NC** | **miR-129-5p** |  |
| *PRPS2* | down | 4248.293 | 563.5657 | -2.91423 |
| *PDK4* | down | 10743.28 | 3466.177 | -1.63202 |
| *HK2* | down | 9880.672 | 3399.653 | -1.53922 |
| *PDP2* | down | 943.4845 | 326.7124 | -1.52998 |
| *LDHAL6B* | down | 37.80148 | 13.7061 | -1.46362 |
| *PGM2* | down | 3371.548 | 1246.236 | -1.43583 |
| *SLC2A3* | down | 328.9966 | 131.1119 | -1.32727 |
| *PDK1* | down | 7336.012 | 3129.293 | -1.22916 |
